# microRNA Expression in Peripheral Blood Cells following Acute Ischemic Stroke and Their Predicted Gene Targets

**DOI:** 10.1371/journal.pone.0099283

**Published:** 2014-06-09

**Authors:** Glen C. Jickling, Bradley P. Ander, Xinhua Zhan, Dylan Noblett, Boryana Stamova, Dazhi Liu

**Affiliations:** Department of Neurology and the MIND Institute, University of California at Davis, Sacramento, California, United States of America; University of Milan, Italy

## Abstract

**Background:**

microRNA (miRNA) are important regulators of gene expression. In patients with ischemic stroke we have previously shown that differences in immune cell gene expression are present. In this study we sought to determine the miRNA that are differentially expressed in peripheral blood cells of patients with acute ischemic stroke and thus may regulate immune cell gene expression.

**Methods:**

miRNA from peripheral blood cells of forty-eight patients with ischemic stroke and vascular risk factor controls were compared. Differentially expressed miRNA in patients with ischemic stroke were determined by microarray with qRT-PCR confirmation. The gene targets and pathways associated with ischemic stroke that may be regulated by the identified miRNA were characterized.

**Results:**

In patients with acute ischemic stroke, miR-122, miR-148a, let-7i, miR-19a, miR-320d, miR-4429 were decreased and miR-363, miR-487b were increased compared to vascular risk factor controls. These miRNA are predicted to regulate several genes in pathways previously identified by gene expression analyses, including toll-like receptor signaling, NF-κβ signaling, leukocyte extravasation signaling, and the prothrombin activation pathway.

**Conclusions:**

Several miRNA are differentially expressed in blood cells of patients with acute ischemic stroke. These miRNA may regulate leukocyte gene expression in ischemic stroke including pathways involved in immune activation, leukocyte extravasation and thrombosis.

## Introduction

microRNA (miRNA) are important regulators of gene expression and play important roles in the initiation and progression of several diseases. Indeed, dysregulation of miRNA in the immune system has been linked to chronic lymphocytic leukemia (miR-15a, miR-16) [1], rheumatoid arthritis (miR-146a) [2], and multiple sclerosis (miR-326) [3]. In stroke miRNA may play an important regulatory role, particularly given the known effects of miRNA on the immune system and vasculature [4]. Preliminary studies indicate miRNA are dysregulated in the blood and brain of rodent ischemic stroke [5]–[7]. However, additional study is required to better understand miRNA in patients with acute ischemic stroke and their regulation of genes and pathways involved in cerebrovascular disease.

miRNA are short (∼17–25 nucleotides long) non-protein coding ribonucleic acids. They regulate gene expression at multiple epigenetic levels including mRNA degradation, mRNA sequestration, translational repression and transcriptional repression [8]. In patients with acute ischemic stroke changes in blood gene expression are present and correspond to differences in the immune and coagulation systems [9]–[12]. Given miRNA are predicted to regulate >60% of known mRNA [13], many of the genes expressed in acute ischemic stroke are likely regulated by miRNA. In this study we sought to determine whether miRNA are differentially expressed in patients with acute cerebral ischemia and thus may be important regulators of leukocyte gene expression in ischemic stroke.

## Methods

### Study Subjects

The study protocol was approved by the University of California Davis Institutional Review Board and all subjects provided informed written consent. Patients were recruited from the University of California Davis from November 2010 to November 2012. There were 24 patients with acute ischemic stroke and 24 vascular risk factor controls. Stroke diagnosis required consensus of two board certified neurologists and restricted diffusion on brain MRI (positive DWI-MRI). Patients with infection (current or within 2 weeks of stroke), immunosuppressive therapy, lymphoma, leukemia, or treatment with thrombolytic therapy were excluded from study. Control subjects had vascular risk factors without prior history of stroke, myocardial infarction or peripheral vascular disease.

### microRNA Isolation

Blood was collected in PAXgene tubes (Pre-AnalytiX/BD) from a venous blood draw performed within 72 hours of stroke onset and stored frozen at −80°C. PAXgene tubes stabilize RNA in blood, which is predominantly from circulating leukocytes (e.g. granulocytes, monocytes, B-cells, T-cells) and megakaryocytes. RNA was isolated according to the manufacturer's protocol (PAXgene blood miRNA kit; Pre-AnalytiX). Quantity and quality of RNA was determined by Nano-Drop (Thermo Fisher) and Agilent 2100 Bioanalyzer (Agilent). Samples required A_260_/A_280_ absorbance ratio ≥1.8, A_260_/A_230_ ≥1.8, 28S/18S rRNA ratio ≥1.8, and an RNA integrity number ≥8.

### microRNA Analysis

microRNA were initially screened by microarray with identified candidates evaluated by qRT-PCR. For the microarray analysis, 200 ng of total RNA was labeled using FlashTag Biotin HSR labeling kits (Affymetrix, CA) with no amplification, hybridized to Affymetrix Gene Chip miRNA 3.0 Arrays, and scanned using an Affymetrix GCS3000 Gene Array Scanner according to manufacturer's protocol (Affymetrix, Santa Clara, CA) (GSE55937). Data were analyzed in Partek Genomics Suite 6.6, normalized using RMA, and log2 transformed. Subjects with acute ischemic stroke were compared to controls with adjustment for microarray batch using ANOVA. miRNA with a fold change >|1.2| and p<0.05 were evaluated by qRT-PCR.

TaqMan microRNA assays (Applied Biosystems, Foster City, CA) were used to evaluate candidate miRNA identified by microarray. Total RNA (25 ng per sample) was converted into cDNA using RT Primers and TaqMan microRNA RT Kit (Applied Biosystems). cDNA was amplified using RT PreAmp Primers and Taqman PreAmp Master Mix (Applied Biosystems). Diluted PreAmp product was mixed with TaqMan Universal PCR Master Mix and Taqman miRNA assay and run using an Applied Biosystems 7900HT real-time PCR instrument (Applied Biosystems). U75 was used as the endogenous control. Cycle thresholds were determined using RQ manager and miRNAs where 95% had a raw cycle threshold score >35 were excluded (Schmittgen et al, 2008). Analysis was performed in Partek Genomics Suite (Partek, St Louis, MI, USA). Differentially expressed miRNAs in ischemic stroke compared to controls had a fold change >|1.5| and a false discovery rate (FDR) corrected p-value<0.05.

### microRNA Targets

mRNA targets of the identified miRNA were determined using target gene databases (Target Scan 6.0 human, Tarbase, miRecords, Ingenuity Pathway Analysis). mRNA were considered targets if there was prior experimentally observed regulation by miRNA or if they were predicted with high probability to be gene targets. mRNA targets of the identified miRNA were compared to genes and pathways associated with human ischemic stroke to evaluate potential miRNA regulation of leukocyte gene expression in ischemic stroke [9]–[12].

## Results

Characteristics of the 24 patients with acute ischemic stroke and 24 control subjects are shown in Table 1. The mean age was 63.1 years and 50% were female. The study population was ethnically diverse with 68.7% Caucasian, 12.5% African American, 6.3% Asian, and 12.5% of other race. No statistically significant differences with respect to demographic or vascular risk factors were present between patients with ischemic stroke and controls (Table 1). The median time from stroke onset to blood draw was 27.4 hours (IQR 10.5–46.0). There were 8 (33%) cardioembolic strokes, 8 (33%) large vessel strokes, and 8 (33%) small vessel lacunar strokes.

**Table 1:** Characteristics of ischemic stroke and vascular risk factor control study subjects.

Characteristic	Control (n = 24)	Ischemic Stroke (n = 24)	p-value
Age years (SD)	63.8 (8.5)	62.4 (8.1)	0.38
Gender male n(%)	12 (50.0%)	12 (50.0%)	1.00
Hypertension History n(%)	21 (87.5%)	18 (75.0%)	0.28
Diabetes History n(%)	8 (33.3%)	7 (29.2%)	0.76
Hyperlipidemia n(%)	16 (66.7%)	16 (66.7%)	1.00
Smoker n(%)	5 (20.8%)	9 (37.5%)	0.20
Atrial Fibrillation n(%)	6 (25.0%)	5 (20.8%)	0.74

**Figure 1:**
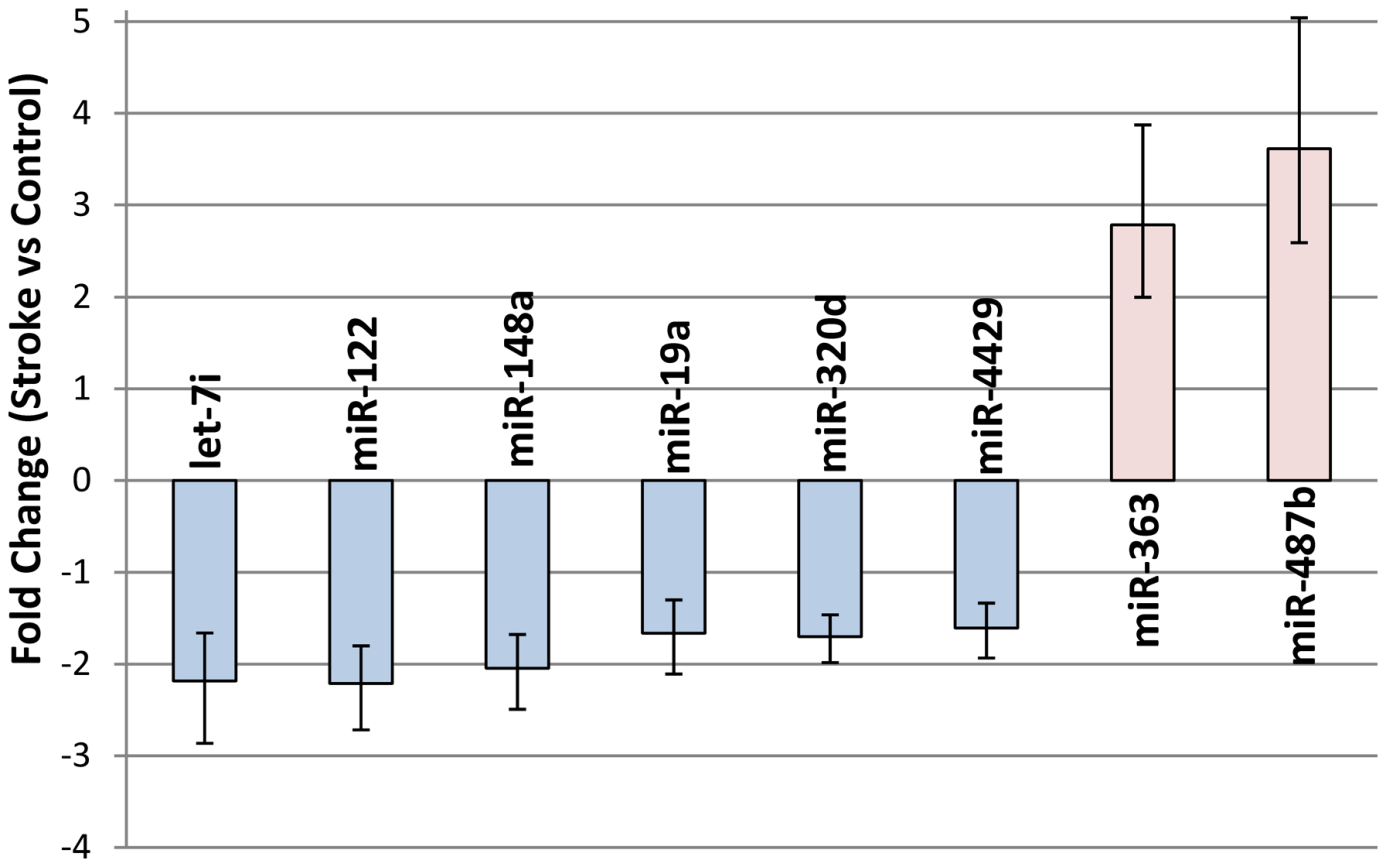
Fold change in microRNA that are significantly differentially expressed in patients with acute ischemic stroke compared to controls with vascular risk factors (FDR<0.05). Error bars indicate standard error. Expression determined by qRT-PCR.

**Figure 2:**
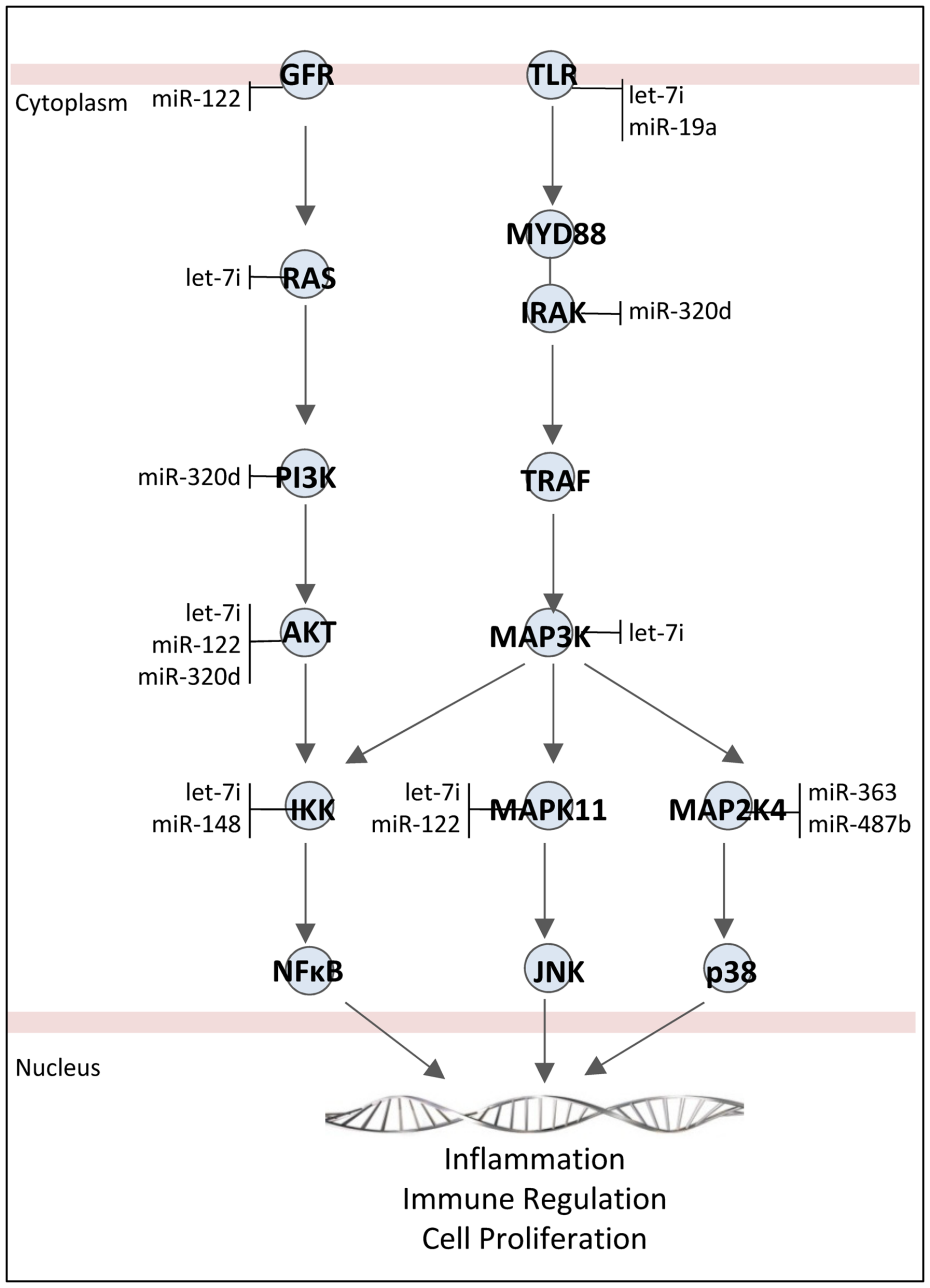
Genes and their microRNA targets in the NF-κB and toll-like receptor signaling pathways. microRNA were identified in the current study as differentially expressed in ischemic stroke, and are predicted to target genes based on prior observation or high predicted probability. Abbreviations: GFR, Growth factor receptor; IKK, Inhibitor of nuclear factor kappa-B kinase; IRAK, Interleukin-1 receptor-associated kinase; MAPK, Mitogen-activated protein kinase; MYD88, Myeloid differentiation primary response 88; PI3K, Phosphatidylinositide 3-kinase; TLR, Toll like receptor; TRAF, TNF receptor associated factor.

There were eight miRNA differentially expressed between acute ischemic stroke and vascular risk factor controls (Figure 1, Figure S1, Table S1). Six miRNA had increased expression miR-122 (FC-2.29, FDR p-value 0.047), miR-148a (FC -2.05, FDR p-value 0.009), let-7i (FC -2.07, FDR p-value 0.023), miR-19a (FC -1.66, FDR p-value 0.030), miR-320d (FC -1.70, FDR p-value 0.020), miR-4429 (FC -1.61, FDR p-value 0.034); and two miRNA had decreased expression miR-363 (FC 3.61, FDR p-value 0.037), miR-487b (FC 2.66, FDR p-value 0.044). These miRNA are either known to regulate or are predicted with high probability to regulate the expression of several genes in pathways associated with ischemic stroke including NF-κβ signaling, toll-like receptor signaling, leukocyte extravasation, interleukin signaling, transforming growth factor-β signaling, and the prothrombin activation pathway (Figure 2, Table S2). At the level of biological systems, several of the miRNA have been shown to regulate aspects of the immune system and coagulation system as presented in the discussion.

## Discussion

We identified miR-122, miR-148a, let-7i, miR-19a, miR-320d, miR-4429 to be decreased and miR-363, miR-487b to be increased in blood cells of patients with acute ischemic stroke. These miRNA may be important regulators of leukocyte gene expression in acute ischemic stroke. They are predicted to regulate a number of genes and pathways associated with ischemic stroke including immune activation, leukocyte extravasation and thrombus formation.

The role of miRNA in patients with ischemic stroke is a developing field, with growing interest for their potential biomarker and therapeutic applications [14]. This report adds to our understanding of miRNA expression and gene regulation in acute cerebral ischemia. Prior studies have reported miRNA expression in patients with subacute and chronic ischemic stroke. Within 7-14 days of stroke onset circulating plasma levels of miR-21, miR-221 [15], and miR-145 [16] are increased whereas miR-210 is decreased [17]. miRNA can also be studied in blood at the intracellular level from circulating leukocytes and other blood cells. In the one stroke study of blood cells, 157 miRNA were reported to be differentially expressed in young patients 6–18 months after stroke when compared to healthy controls [18]. In the current study we report the cellular blood miRNA expressed in patients with acute ischemic stroke. Several of the identified miRNA in acute cerebral ischemia overlap with those reported in subacute and chronic stroke including let-7i, miR-19a, miR-320d, miR-363, and miR-487b. However, additional study is required to delineate the time course of miRNA expression following ischemic stroke and the relationship between plasma and cellular blood miRNA.

### miRNA Regulation of Leukocyte Activation

miRNA may regulate activation of the immune system in ischemic stroke. Following ischemic brain injury, peripheral leukocytes are activated via release of cytokines, chemokines, and Damage Associated Molecular Pattern molecules (DAMPs). We previously have found that genes involved in TLR signaling and NFKB signaling are differentially expressed in patients with acute ischemic stroke [9]–[12]. Several of the cellular blood miRNA identified act on genes in these pathways including let-7i, miR-19a, miR-320 (Figure 2). These miRNA may modulate immune activation and inflammatory response following stroke. Indeed, several of these miRNA have a known biological effect on leukocytes. Let-7i regulates toll-like receptor signaling in monocytes, modulates the differentiation of dendritic cells, and directs T-cells toward a Th1 phenotype [19], [20]. miR-122 regulates the expression of peroxiredoxin 2, a DAMP involved in immune activation post stroke [21]. miRNA-148 fine tunes immune response by altering cytokine production (IL6, TNF-a, IL-12, TNFSF7), T-cell proliferation, MHC II expression, and dendritic cell antigen presentation [22], [23]. miR-19b is reported to negatively regulate inflammation in humans and activate expression of TLR2 and TLR4 [24], [25]. Thus miR-19a may promote inflammatory response in ischemic stroke.

### miRNA Regulation of Leukocyte Extravasation

miRNA may also regulate genes involved in leukocyte extravasation following ischemic stroke. We previously have found that genes involved in leukocyte adhesion and blood brain barrier disruption (MMPs) are differentially expressed in patients with acute ischemic stroke [9]–[12]. Of the cellular blood miRNA identified, several may regulate these genes. miR-148 is known to regulate leukocyte expression of the adhesion molecule LFA-1 and the matrix metalloproteases MMP10, MMP13, and MMP-15 [23]. miR-487b family members facilitate cellular invasion by up-regulating MMPs in myeloid cells [26]. Of note, miR-487b is induced by hypoxia [27]. miR-320 regulates ischemic-reperfusion injury in cardiac tissue and inhibiting miR-320 reduces infarct size in myocardial ischemia [28]. miR-320 targets ETS2, a transcription factor that regulates the expression of several genes important to leukocyte extravasation post-stroke including MMP-9, MMP-3, MMP-13, ANGPT1, and TNF [29], [30]. miRNA-19a regulates MMP-3 via TLR2 [24] and has angiogenic roles that may contribute to blood brain barrier breakdown and vascular remodeling post stroke [31].

### miRNA Regulation of Thrombus Formation

Aspects of thrombus formation may also be regulated by miRNA in ischemic stroke. miRNA are known to regulate coagulation and platelets [32], [33]. Tissue factor is a central component in the coagulation cascade and monocytes are a major source. miR-19a acts on tissue factor pathway inhibitor (TFPI) to reduce tissue factor activity and prevent clot formation. Thus, in ischemic stroke miR-19a may modulate thrombus formation through effects on TFPI. Another gene that may be regulated by miRNA in ischemic stroke is SERPINE1 (plasminogen activator inhibitor-1 (PAI-1). SERPINE1 codes for the principal inhibitor of tissue plasminogen activator (tPA) and urokinase (uPA), which act on plasminogen to promote fibrinolysis. miR-148a and miR-19a both target SERPINE1 and may regulate the activity of endogenous tPA in ischemic stroke. Other coagulation factors that may be regulated by miRNA in ischemic stroke include thrombin (let-7i), coagulation factor 8 (miR-122), and coagulation factor 3 (miR-148, miR-19a).

Strengths of this study include a comparison of ischemic stroke patients to controls similar in age and vascular risk factors, evaluation of all known miRNA by microarray with qRT-PCR confirmation, and the use of PAXgene tubes which reduce changes that occur as a result of RNA degradation. This preliminary study has limitations. Sample size remains small; thus additional studies in larger cohorts using multiple methods to measure and confirm miRNA expression are required. This study focused on all ischemic stroke. However, certain miRNA may be differentially expressed by subtype of ischemic stroke, which will require a larger cohort to examine. The gene targets of the miRNA were identified using target databases. Additional studies are required to experimentally evaluate miRNA gene targets and determine their functional role in ischemic stroke.

In conclusion, several miRNA are differentially expressed in blood cells of patients with acute ischemic stroke. These miRNA are predicted to regulate gene expression and pathways in blood that are associated with ischemic stroke. With additional study, the identified miRNA may serve as markers of cerebral ischemia or provide novel methods to modulate the immune and coagulation systems in ischemic stroke.

## Supporting Information

Figure S1 Relative microRNA expression in patients with acute ischemic stroke compared to vascular risk factor controls. Expression determined by qRT-PCR relative to U75, with lowest expressing miRNA assigned a value of 1.
(TIF)

Table S1 microRNA differentially expressed between acute ischemic stroke and vascular risk factor controls.
(PDF)

Table S2 Pathways previously associated with ischemic stroke by gene expression analysis of blood and the microRNA targeting genes in that pathway. Gene targets are experimentally observed or predicted with high probability to be targets.
(PDF)
