# Supplementary material for: microRNA Expression in Peripheral Blood Cells following Acute Ischemic Stroke and Their Predicted Gene Targets
Source: PLoS One. 2014 Jun 9;9(6):e99283. doi: 10.1371/journal.pone.0099283 (PMC4050059; doi:10.1371/journal.pone.0099283)
Supplement: Table S1 — microRNA differentially expressed between acute ischemic stroke and vascular risk factor controls. (PDF) [file pone.0099283.s002.pdf]

Table S1: microRNA differentially expressed between acute ischemic stroke and vascular risk factor controls.

		qRT-PCR			Microarray		
**microRNA**	**Sequence**	**Detector (ABI Taqman Assay ID)**	**FDR p-value**	**Fold Change (Stroke vs Control)**	**Affymetrix Probeset ID**	**p-value**	**Fold-Change**
let-7i-5p	UGAGGUAGUAGUUUGUGCUGUU	hsa-let-7i-002221	0.023	-2.07	hsa-let-7i_st	0.044	-1.46
miR-122	UGGAGUGUGACAAUGGUGUUUG	hsa-miR-122-002245	0.047	-2.29	hsa-miR-122_st	0.011	-1.86
miR-148a	UCAGUGCACUACAGAACUUUGU	hsa-miR-148a-000470	0.009	-2.05	hsa-miR-148a_st	0.021	-1.46
miR-19a	UGUGCAAAUCUAUGCAAAACUGA	hsa-miR-19a-000395	0.030	-1.66	hsa-miR-19a_st	0.040	-1.45
miR-320d	AAAAGCUGGGUUGAGAGGA	hsa-miR-320d-241066_mat	0.020	-1.70	hsa-miR-320d_st	0.022	-1.29
miR-4429	AAAAGCUGGGCUGAGAGGCG	hsa-miR-4429-464083_mat	0.034	-1.61	hsa-miR-4429_st	0.048	-1.26
miR-363-3p	CGGGUGGAUCACGAUGCAAUUU	hsa-miR-363*-001283	0.037	3.61	hsa-miR-363-star_st	0.014	1.29
miR-487b	AAUCGUACAGGGUCAUCCACUU	hsa-miR-487b-001285	0.044	2.66	hsa-miR-487b_st	0.036	1.37
