# Supplementary material for: microRNA Expression in Peripheral Blood Cells following Acute Ischemic Stroke and Their Predicted Gene Targets
Source: PLoS One. 2014 Jun 9;9(6):e99283. doi: 10.1371/journal.pone.0099283 (PMC4050059; doi:10.1371/journal.pone.0099283)
Supplement: Table S2 — Pathways previously associated with ischemic stroke by gene expression analysis of blood and the microRNA targeting genes in that pathway. Gene targets are experimentally observed or predicted with high probability to be targets. (PDF) [file pone.0099283.s003.pdf]

Table S2: Pathways previously associated with ischemic stroke by gene expression analysis of blood and the microRNA targeting genes in that pathway. Gene targets are experimentally observed or predicted with high probability to be targets.

Pathway	miRNA	Gene Targets
**Toll Like Receptor Signaling**	let-7i-5p	AKT2, CCL3, CCL7, CD86, IKK-α, IL10, IL6, MAP3K1, MAP4K4, MAPK11, PPARA, TAB2, TLR4, TNFAIP3
	miR-122-5p	AKT3, CD83, ELK1, MAPK11, NOD2
	miR-148a	IKK-α, IKBKB, ITGA5
	miR-19a	PPARA, TLR5, TNF, TNFAIP3
	miR-320d	AKT3, GRB2, IRAK2, IRAK3, MAPK1, PLCG1
	miR-363-3p	ITGA5, MAP2K4
	miR-487b	MAP2K4
**NF-κB Signaling**	let-7i-5p	AKT2, IKK-α, GHR, HRAS, IGF1R, INSR, KRAS, MAP3K3, MAP4K4, NGF, NRAS, NTRK3, Ras, TAB2, TGFBR1, TGFBR3, TLR4, TNFAIP3, TNFRSF1B
	miR-122-5p	AKT3, CD40LG, IGF1R, IL1RN, PELI1
	miR-148a	IKK-α, IKBKB, TGFA
	miR-19a	AKT, CARD10, CASP8, GSK3B, TBK1, TNF, TNFA1P3, TNIP1
	miR-320d	AKT3, BMPR1A, IGF1R, IRAK3, FADD, PIK3R1, TGFBR1, TGFBR2
	miR-363-3p	TNF
**Leukocyte Extravasation Signaling**	let-7i-5p	MLLT4, MMP11, RASGRP1
	miR-122-5p	CXCR2, RASSF5
	miR-148a	GLG1, MMP10, MMP13, MMP15, MAP3K4
	miR-19a	CXCL12, ILF3, IL1R1, PTK2B, TNF
	miR-320d	ITGB3, MMP16, MMP19, RAC1
	miR-363-3p	CDC42, MMP16, VCL
**Interleukin-1 Signaling**	let-7i-5p	MAP3K1, TAB2
	miR-122-5p	GNG13, MAPK11
	miR-19a	IL1R1, MAP3K14
	miR-320d	GNAI1, GNAZ, IL1RAP, IRAK2, IRAK3, MAPK1, MAPK9, PRKAG2, PRKAR1A
	miR-487b	MAP2K4
**Interleukin-6 Signaling**	let-7i-5p	IL6, IL6R, COL1A1, SOCS1, CYP19A1, Ras
	miR-122-5p	AKT3, ELK1, IL1RN, MAPK11
	miR-148a	IL6ST, SOCS3
	miR-19a	IL6ST, RAF, SOCS1, SOCS3, TNF
	miR-320d	AKT3, GRB2, IL6R, JAK2, MAPK1, MAPK9, MCL1, PIK3R1
	miR-363-3p	IL6ST, MCL1, RAF1
	miR-487b	MAP2K4
**TGF-β Signaling**	let-7i-5p	Ras
	miR-122-5p	MAPK11, RUNX2
	miR-148a	SERPINE1
	miR-19a	GSC, PMEPA1, RAF1, RNF111, RUNX3, SERPINE1, SMAD4, SMURF1, TGIF1,
	miR-320d	ACVR2B, BMPR1A, GRB2, MAPK1, MAPK9, SMAD4, SMURF1, TGFBR1, TGFBR2
	miR-363-3p	CDC42, MAP2K4, PMEPA1, RAF1, SK1, SMAD6, SMAD7, SMURF1, TG1F1
	miR-487b	MAP2K4
**Chemokine Signaling**	let-7i-5p	CCL7, HRAS, KRAS, NRAS, RAS
	miR-122-5p	CALM1, MAPK11, CFL1, GNAI3, GNAQ, KRAS, MAP2K1, MRAS, PLCB4, PLCG1,
	miR-19a	CXCL12, PTK2B, RAF1
	miR-320d	CALM1, GNAI1, MAPK1, PLCG1, PPP1CB
	miR-363-3p	RAF1
	miR-487b	CXCR4
**Prothrombin Activation Pathway**	let-7i-5p	COL1A1, COL1A2, COL3A1, F2
	miR-122-5p	F8
	miR-148a	SERPINE1, F3
	miR-19a	SERPINE1, F3, TFPI
